# Randomized Comparison of the Therapeutic Effect of Acupuncture, Massage, and Tachibana-Style-Method on Stiff Shoulders by Measuring Muscle Firmness, VAS, Pulse, and Blood Pressure

**DOI:** 10.1155/2012/989705

**Published:** 2012-02-25

**Authors:** Kazuhiro Tachibana, Noriyuki Ueki, Takuji Uchida, Hiroshi Koga

**Affiliations:** ^1^Nihon Traditional Medical Society, Denenchofu 2-45-8 Ota-ku, Tokyo 145-0071, Japan; ^2^Shinjuku Vocational College of Acupuncture, Moxibustion and Judo Therapy (SSJS), Samoncho 5, Shinjuku-ku, Tokyo 160-007, Japan; ^3^Department of Epidemiology and Public Health, Tokyo Dental College, Mihama-ku, Chiba 261-8502, Japan

## Abstract

To compare the therapeutic efficacy of acupuncture, massage, and Tachibana-Ryojutsu (one of Japanese traditional body balance therapy techniques (SEITAI)), on stiff shoulders, the subjects' muscle firmness, blood pressure, pulse, VAS, and body temperature were measured before and after the treatment. Forty-seven volunteer subjects gave written informed consent to participate in this study. The subjects were randomly divided into three groups to receive acupuncture, massage, or Tachibana-Ryojutsu. Each therapy lasted for 90 seconds. The acupuncture treatment was applied by a retaining-needle at GB-21, massage was conducted softly on the shoulders, and Tachibana-Ryojutsu treated only the muscles and joints from the legs to buttocks without touching the shoulders or backs. The study indicated that the muscle firmness and VAS of the Tachibana-Ryojutsu group decreased significantly in comparison with the acupuncture and massage groups after treatment.

## 1. Introduction

Recently alternative medical treatments have become increasingly popular. In Japan, acupuncture, massage, body balance therapy, and chiropractic are commonly practiced [1]. These treatments effectively treat nerve and musculoskeletal systems, including stiff shoulders. According to the Ministry of Health and Welfare of Japan, stiff shoulders are the most common musculoskeletal complaint among women and the second largest complaint among all people. Many patients are unsure of the most effective type of therapy because the cause of stiff shoulders has not been proven scientifically. Many practitioners may also be unaware of the most effective type of therapy. Therefore, we conducted this study to compare the most commonly practiced therapies for stiff shoulders—acupuncture, massage, and body balance therapy. We have chosen Tachibana-style-method for body balance therapy, one of many body therapy methods in Japan.

## 2. Subjects and Methods

### 2.1. Subjects

We recruited volunteers who were healthy and had stiff shoulder disorder among students, lecturers, and affiliates of Shinjuku Vocational College of Acupuncture, Moxibustion and Judo Therapy (SSJS). As a result, 47 people, 29 males and 18 females, joined the examination. Their average age was 36.1 (standard deviation of 9.8). The subjects received an explanation of the process and signed their informed consent to take part in the examination. Then, they were randomly assigned to one of the three groups, the acupuncture group, the massage group, or the Tachibana-Ryojutsu group by a computer application. Microsoft Excel 2007 was used for the randomization. As a result, 15 were assigned to the acupuncture group, 15 to the massage group, and 17 to the Tachibana-Ryojutsu group. The subjects were not informed which treatment they would receive until just before the examination. The research was conducted after being approved by the Research and Ethics Panel of SSJS in May 2010.

### 2.2. Methods

The research was conducted between September 2 and October 28, 2010, in an experimental laboratory of SSJS at the room temperature (26 ± 0.5 degree Celsius). Each treatment for all subjects was conducted by a single practitioner in order to prevent the variabilities that could be caused by different practitioners. The muscle firmness of all subjects was measured by a single person who did not conduct the treatments.

The subjects were informed which of the three treatments he or she would receive among the three just before the examination. They were only examined after confirming they were healthy, were without chronic disease, and had not experienced side effects after treatment in the past.

Subjects were asked to describe the stiffness in their shoulders by using the Visual Analog Scale (VAS) on the scale of 0 to 10 by checking on a 10 cm line: 0 indicates no stiffness and 10 indicates the strongest stiffness.

The subjects' blood pressure, pulse, and VAS were measured both before and after the treatment in sitting. The subjects' muscle firmness was measured lying face down positions on a care bed. The treatment was conducted in the same position.

**Figure 1:**
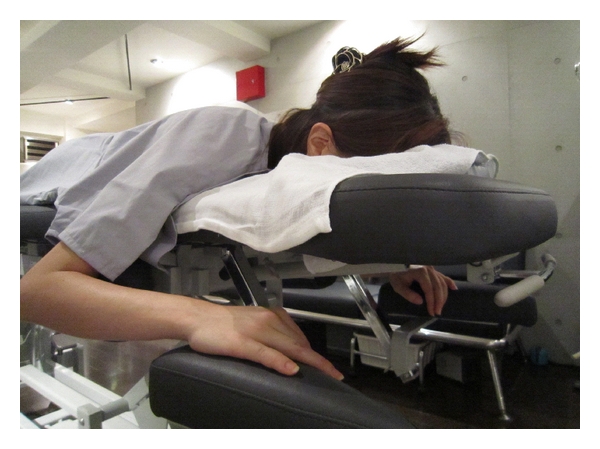


In the face-down position, the subjects' upper arms (from shoulders to elbow) hung straight down at a 90-degree angle from the shoulder, while the forearms (from elbow to hand) were bent at a 90-degree angle to be almost parallel to the chest and allowed to rest on a shelf (Figure 1).

We carefully arranged the subjects' physical movements as to not affect the measured value. It was decided that the muscle firmness and the body temperature would be taken at GB-21, which is located in the middle of the spinous process of vertebra prominens and the acromion. Both sides were measured by a scale, and the precise location was decided. 

We also measured the temperature of the body surface at GB-21.

The muscle firmness was measured 5 times consecutively at GB-21, both before and after the treatment. The largest and smallest readings were discarded and the remain three readings averaged to create the final value.

Treatment was performed for 90 seconds only on the left side of a subjects' body. No treatment was done on the right side of a body.

1. For the 15 subjects in the acupuncture group, the area around GB-21 was cleansed with ethanol. A disposable needle made by Seirin Co. Ltd. (4.0 mm long, 0.18 mm in diameter) was inserted vertically to the skin surface for 1 cm at the measurement point and left there. After taking the needle out, the area around GB-21 was cleansed with ethanol again.
2. For the 15 subjects in the massage group, a towel was placed over the GB-21 area and massage conducted.
3. For the 17 subjects in the Tachibana-Ryojutsu group, the shoulder areas of subjects were not touched. The muscle and joint adjustment was conducted only from the leg to the buttock.

### 2.3. Equipment

At this examination, a digital automated sphygmomanometer (HEM-759P Fuzzy, Omron Corporation, Tokyo) was used to measure the blood pressure, a digital thermometer (model: TH-200, Suzuki Iryoki, Tokyo) was used to measure the temperature of surface of a body, and a neutone meter (Neutone TDM-NA1, TRY-ALL Ltd.) was used for the muscle firmness.

### 2.4. Statistical Analysis

Using Statcel 2 by OMS Publishing to analyze the data of the three groups, One-way factorial ANOVA without replication and multiple comparison were conducted. Furthermore, in order to review the reliableness of the muscle firmness measuring equipment, Intraclass Correlation Coefficient (ICC) was derived by a formula, calculated by Microsoft Excel 2007.

## 3. Results

### 3.1. The Muscle Firmness on the Treated Side: The Change of the Muscle Firmness before and after the Treatment

**Figure 2:**
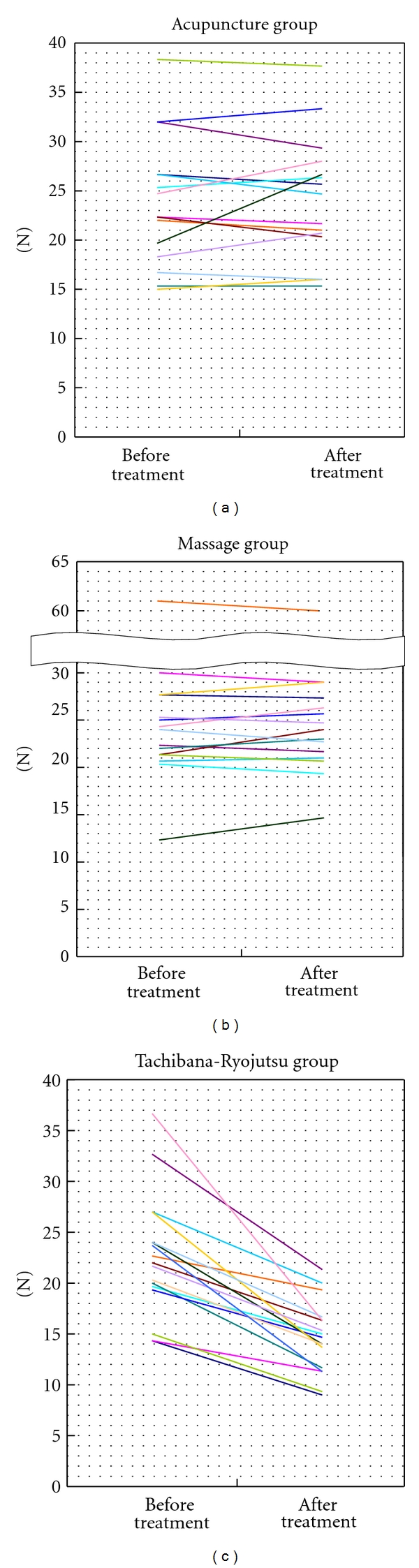
Muscle firmness 90 seconds before and after the treatment on the treated side (left).

Among the 15 subjects in the acupuncture group, muscle firmness rose on 6 people, decreased on 8, and did not change for 1 person. Among the 15 subjects in the massage group, muscle firmness rose for 7 and decreased for 8. The muscle firmness decreased on all 17 subjects in the Tachibana-Ryojutsu group (Figure 2).

We conducted one-way analysis of variance, and there was no distributional difference in all groups.

When the three groups of the muscle firmness on the treated side before and after the treatment were compared, the difference of muscle firmness of the Tachibana-Ryojutsu group (i.e., the amount of decrease) was more significant than in the other groups (*P* < 0.01). However, there was no significant difference between the acupuncture group and the massage group.

### 3.2. The Muscle Firmness of Untreated Area: The Change of the Muscle Firmness before and after the Treatment

Among the 15 subjects in the acupuncture group, muscle firmness rose on 3 people, decreased on 8, and did not change on 4. Among the 15 subjects in the massage group, muscle firmness rose on 6 and decreased on 9. Among the 17 subjects in the Tachibana-Ryojutsu group, it rose on 6, decreased on 10, and did not change on 2.

We conducted one-way analysis of variance, and there was no distributional difference in all groups.

When the muscle firmness on the untreated side before and after the treatment was compared, the difference of the muscle firmness in the Tachibana-Ryojutsu group, or the amount of decrease, was more significant than the massage group (*P* < 0.05). However, there was no significant difference between the acupuncture group and the massage group, and between the acupuncture group and the Tachibana-Ryojutsu group.

### 3.3. The Change of Visual Analog Scale (VAS): The Change of Visual Analog Scale (VAS) before and after the Treatment

**Figure 3:**
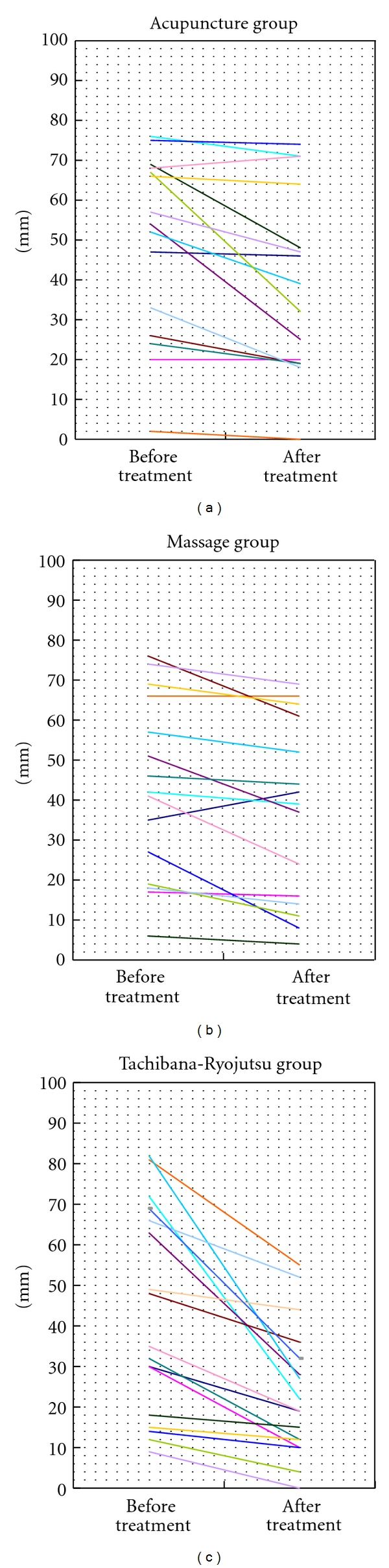
The change of Visual Analog Scale after 90 seconds.

Among the 15 subjects in the acupuncture group, a rise in the VAS occurred for 1 person, a decrease occurred for 13, and 1 felt no change. Among the 15 subjects in the massage group, a rise occurred for 1, a decrease occurred for 13 and 1 felt no change. Among the 17 subjects in the Tachibana-Ryojutsu group, the VAS decreased for all (Figure 3).

We conducted one-way analysis of variance, and there was no distributional difference in all groups.

When the three groups of VAS before and after the treatment were compared, the Tachibana-Ryojutsu group had significantly more palliative efficacy than the massage group (*P* < 0.05). However, there was no significant difference between the Tachibana-Ryojutsu group and the acupuncture group, as well as the acupuncture group and the massage group.

### 3.4. The Change of the Pulse

Among the 15 subjects in the acupuncture group, a rise of pulse was found in 6 people, a decrease in 7, and no change in 2. Among the 15 subjects in the massage group, a rise was found in 2, a decrease in 10, and no change in 3. Among the 17 subjects in the Tachibana-Ryojutsu group, pulse rose for 1, decreased for 14, and did not change for 2.

We conducted one-way analysis of variance, and there was no distributional difference in all groups. 

When the pulses before and after the treatment were compared, there was no significant difference between the three groups according to Sheffe's test. However, when they are compared with the Tukey-Kramer method, the changes in pulses in the Tachibana-Ryojutsu group were more significant than those in the acupuncture group (*P* < 0.05). There were no significant differences between the acupuncture group and the massage group, and the massage group and the Tachibana-Ryojutsu group.

### 3.5. Systolic Arterial Pressure

When we compared the systolic arterial pressure before and after the treatment of the three groups, there was no statistically significant difference.

By one-way analysis of variance, there was no distributional difference in all groups.

### 3.6. Diastolic Blood Pressure

When we compared the Diastolic blood pressure before and after the treatment of the three groups, there was no statistically significant difference.

By one-way analysis of variance, there was no distributional difference in all groups.

### 3.7. Body Temperature on the Treated Side: The Change of the Body Temperature before and after the Treatment on the Treated Side (Left)

Among the 15 subjects in the acupuncture group, a rise of body temperature was found in 4 people and a decrease in 11. Among the 15 subjects in the massage group, a rise was detected in 11, a decrease in 3, and no change was evident in 1 person. Among the 17 subjects in the Tachibana-Ryojutsu group, a rise was found in 4 and a decrease in 13.

We conducted one-way analysis of variance, and there was no distributional difference in all groups.

When the body temperatures before and after the treatment were compared, the body temperature in the massage group was more statistically significantly raised than the ones in the acupuncture and the Tachibana-Ryojutsu groups (*P* < 0.05). However, there were no significant differences between the acupuncture group and the Tachibana-Ryojutsu group.

### 3.8. Body Temperature on the Untreated Side

When we compared the body temperature on the untreated side before and after the treatment of the three groups, there was no statistically significant difference. 

By one-way analysis of variance, there was no distributional difference in all groups.

### 3.9. The Conclusion of the Examination Result

See Table 1 for the conclusion of the examination result.

## 4. Discussion

### 4.1. The Validity of Experimental Maneuver

#### 4.1.1. The Confidence of the Value of Muscle Firmness

 Palpating muscle firmness on the surface of a body is an important method not only for traditional medical treatment but also for modern scientific medicine, like physical therapy. Palpation is also used often to diagnose and judge the efficacy of a cure. However, when muscle firmness is measured by the practitioner's palpation subjectively, it was not possible to accept it as objective data. Recently, several kinds of simple muscle firmness indicators have been developed in order to use the data as a barometer of muscle tension [2]. For this study, we used the muscle firmness indicator TDM-N1 developed by Try-all Corporation. Tomoko Koeda and others have examined the adequacy, repeatability and clinical usability of this indicator. When they used a number of individuals to measure muscle firmness of one subject, the results varied, but when a single person measured all of the subjects, the result was stable enough to use as a barometer of relative comparison [2]. Additionally, Saito and others state that it is important to give vertical pressure to the muscle precisely and stably when a muscle firmness indicator is used [3].

Based upon these reports, in this study one individual measured the subjects. The measurer was trained for three months to give vertical pressure precisely and stably at GB-21 to provide consistency. In addition, in order to measure the muscle firmness before and after the treatment precisely, we minimized the margin of error by having all the subjects assume the same body position (in the face-down position) and the same foot position. The interclass correlation coefficient (ICC) of one-way analysis of variance of the muscle firmness value that we applied for this examination proved of high authenticity of ICC (1,3) = 0.982. Thus, we have confirmed the high repeatability and efficiency of the muscle firmness indicator used in this study.

#### 4.1.2. The Interposing Time, 90 Seconds

 During this study, in order to limit the physical and psychological effects caused by a recumbent position, we shortened the treatment to 90 seconds. During usual acupuncture treatments, needles are generally placed at multiple spots and left in place between 5 and 20 minutes. However, a study conducted by Ceccherelli and others found that acupuncture's effects on neck-shoulder area appeared in a relatively short time, and the number of the stimulus needles was not correlated with the effectiveness of treatment [4]. Following this report, we set up the time of 90 seconds for the acupuncture treatment group at this examination. Thus, the efficacy of each treatment can be easily compared per unit of time.

#### 4.1.3. The GB-21 Point for Shoulder Stiffness

GB-21 is a pressure point that is often used to treat shoulder stiffness. Therefore, we used the spot for this study.

### 4.2. The Muscle Firmness and VAS (Visual Analog Scale) at Each Treatment Group

#### 4.2.1. The Acupuncture Group

In this study, acupuncture failed to show a significant decrease in the muscle firmness, but VAS did decrease after treatment. Five subjects exhibited a decrease in VAS but a rise in muscle firmness. There are two explanations for this. First, the use of acupuncture to control pain is well known and some articles have reported that it can alleviate muscle pain [5–7]. It is possible that the subjective VASs of these five subjects were lowered because they assumed that the acupuncture treatment would work. Second, it is also possible that acupuncture did not produce a significant difference in this study because the examination was conducted under different conditions from those studies that showed a significant difference. These different conditions are stimulation time, stimulation spot, and stimulation means (conduction, nonconduction, etc.).

#### 4.2.2. The Massage Group

The massage treatment also lowered the average VAS but did not lower muscle firmness. However, a greater rise in body temperature was observed than in the other treatment groups. Massage treatment aims at improving circulation of the periphery by mechanical stimulus on the body surface. This effect was observed as a rise in the body temperature and the improvement of VAS. Tomoko Koeda and others evaluated the efficiency of the massage on the triceps surae muscle, and they found that the muscle firmness was more significantly lowered than in a control group [2]. However, in this examination, the muscle firmness was not lowered. As tension of trapezius muscle also can be caused by Viscero-Cutaneous reflex, massaging the trapezius muscle may have been ineffective on these subjects as the point of tension derived from the Viscero-Cutaneous reflex.

#### 4.2.3. The Tachibana-Ryojutsu Group

The muscle firmness and VASs of all the subjects in the Tachibana-Ryojutsu group were lowered. The muscle firmness on the treated side was significantly lowered compared to the other groups (*P* = 2.18 × 10^−10^), and VAS was also lowered significantly compared with the other groups. The muscle firmness on the treated side of none of the subjects after the Tachibana-Ryojutsu was raised. VASs of all 17 subjects were lowered. This suggests that the treatment alleviated muscle pain and muscle stiffness with high repeatability. It is interesting that, with the Tachibana-Ryojutsu method, the practitioner did not touch the shoulder and back area, and the treatment was only through the muscle adjustment and exercise from the leg to buttocks. Furthermore, the muscle firmness on the untreated side (the right side) was lowered (*P* < 0.05) in 10 of the 17 subjects. It is possible this was caused by the influence of the treatment on the untreated side kinematically, or by commissure reaction or neurological reaction. The Tachibana-Ryojutsu showed higher efficiency per unit of time by lowering the muscle firmness and VAS than the other two groups.

### 4.3. The Relationship between the Muscle Firmness and VAS

The subjects in the acupuncture and massage groups were treated directly at the point of shoulder stiffness, and their pain and complaints were only eased, while the Tachibana-Ryojutsu, which was conducted remotely, produced a marked reduction of pain and muscular strain. Harumi Kogo and others examined the relationship between the muscle stiffness and muscle pain using people who had stiff shoulders. They found that there was no strong correlation. However, muscle pain can be caused by hypertonic muscle and induration of muscle for an extended period of time [8]. Therefore, it is possible that there is no pain even if the muscle is hypertonic condition if the condition does not last long enough.

### 4.4. The Effect on the Circulatory System by the Tachibana-Ryojutsu

In this examination, the Tachibana-Ryojutsu significantly lowered the pulse compared to acupuncture (*P* < 0.05). This suggests that the Tachibana-Ryojutsu influenced the subjects' circulatory system or autonomic nervous system. The possible mechanisms affecting the autonomic nervous system are the following.

Nishijo and others define that when the autonomicity of the heart is 100, the pulse at rest (*z*) is *Z* = 100 + *Y* − *X* (*Y*: sympathetic participation late, *X*: parasympathetic participation late) [9]. That is to say, the pulse at rest is proportional to the sympathetic participation rate while it is inversely proportional to the parasympathetic participation rate. 

If this relationship is applied to this examination, regarding the decrease of the pulse, the Tachibana-Ryojutsu worked to lower the involvement of the sympathetic nerve or to raise the one of the parasympathetic participation rate. Both may have happened at the same time.

The interposition to autonomic nervous system appeared here more significantly than the acupuncture group, so it is considered that the Tachibana-Ryojutsu system not only alleviates muscle stiffness but also works on the medical problems.

## 5. Conclusions

The study showed that Tachibana-Ryojutsu, which stimulated distant areas and didnot touch the lesion, outperformed acupuncture therapy and massage that were directly applied to the lesion. This suggests that the existence of a route from the distant part to the lesion has been proven statistically. The investigation indicated that the Tachibana-Ryojutsu made the subjects more comfortable by relieving their stiff shoulders better than massage and acupuncture.

## Figures and Tables

**Table 1:** The conclusion of the examination result.

Indicators	Ac versus Ma	Ac versus T-R	Ma versus T-R
The muscle firmness on the treated side	NS	**(T-R)	**(T-R)
The muscle firmness of untreated area	NS	NS	*(T-R)
VAS	NS	NS	*(T-R)
Pulse	NS	O (T-R)	NS
Systolic blood pressure	NS	NS	NS
Diastolic blood pressure	NS	NS	NS
The body temperature at the spot on the treated side	*(Ma)	NS	*(Ma)
The body temperature at the spot on the untreated side	NS	NS	NS

*: significant difference by Scheffe's *F* test (*P* < 0.05).

**: significant difference by Scheffe's *F* test (*P* < 0.01).

O: significant difference by Turkey-Kramer method (*P* < 0.05).

NS: no significant difference.

Ac: Acupuncture; Ma: Massage; T-R: Tachibana-Ryojutsu.
